# RTN and Annealing Related to Stress and Temperature in FIND RRAM Array

**DOI:** 10.1186/s11671-018-2846-1

**Published:** 2019-01-08

**Authors:** Chih Yuan Chen, Chrong Jung Lin, Ya-Chin King

**Affiliations:** Institute of Electronics Engineering, National Tsing Hua University, Hsinchu, Taiwan

**Keywords:** Random telegraph noise, Advanced FinFET technology, RRAM, Anneal

## Abstract

In this work, an observation on random telegraph noise (RTN) signal in the read current of a FinFET dielectric RRAM (FIND RRAM) device is presented. The RTN signal of a FIND RRAM cell is found to change after the device being subjected to cycling stress. After undergoing cycling stress, RRAM cells have a stronger tendency to show more frequent and intense RTN signals. The increase of noise levels in FIND RRAM cells can be alleviated generally by high temperature anneal, and with this concept, an on chip annealing scheme is proposed and demonstrated.

## Introduction

Continuous scaling of CMOS technology improved the characteristics and performance of integrated circuits drastically in the past decade. However, as the technology node is scaled down below 20 nm, variations due to single atom/electron in device characteristics increases, for example, random dopant fluctuations (RDF), and thus bringing forth fundamental issues that cannot be overseen [1]. For instance, any variations in the number of carriers or structural defects have a much larger impact on the output and performance in a scaled device, and the effects of device scaling on variability due to RDF and gate line-edge roughness (LER) have also been reported [2–4]. Random telegraph noise (RTN) is thought to be another major challenge for devices with small area, such as NAND Flash and RRAMs [5–11]. In this work, we investigate the RTN noise in an n-channel FinFET-based FIND RRAM cell, which has already been successfully implemented in standard logic process in 1kbit arrays [12]. Changes in the RTN in response to cycling stresses and high-temperature bake are observed. In this work, the effects of stress and temperature on the RTN noise in FIND RRAM cells is studied, and an on-chip annealing scheme is proposed to alleviate the after cycling time-variant read current noise.

## Background and Methods

**Fig. 1:**
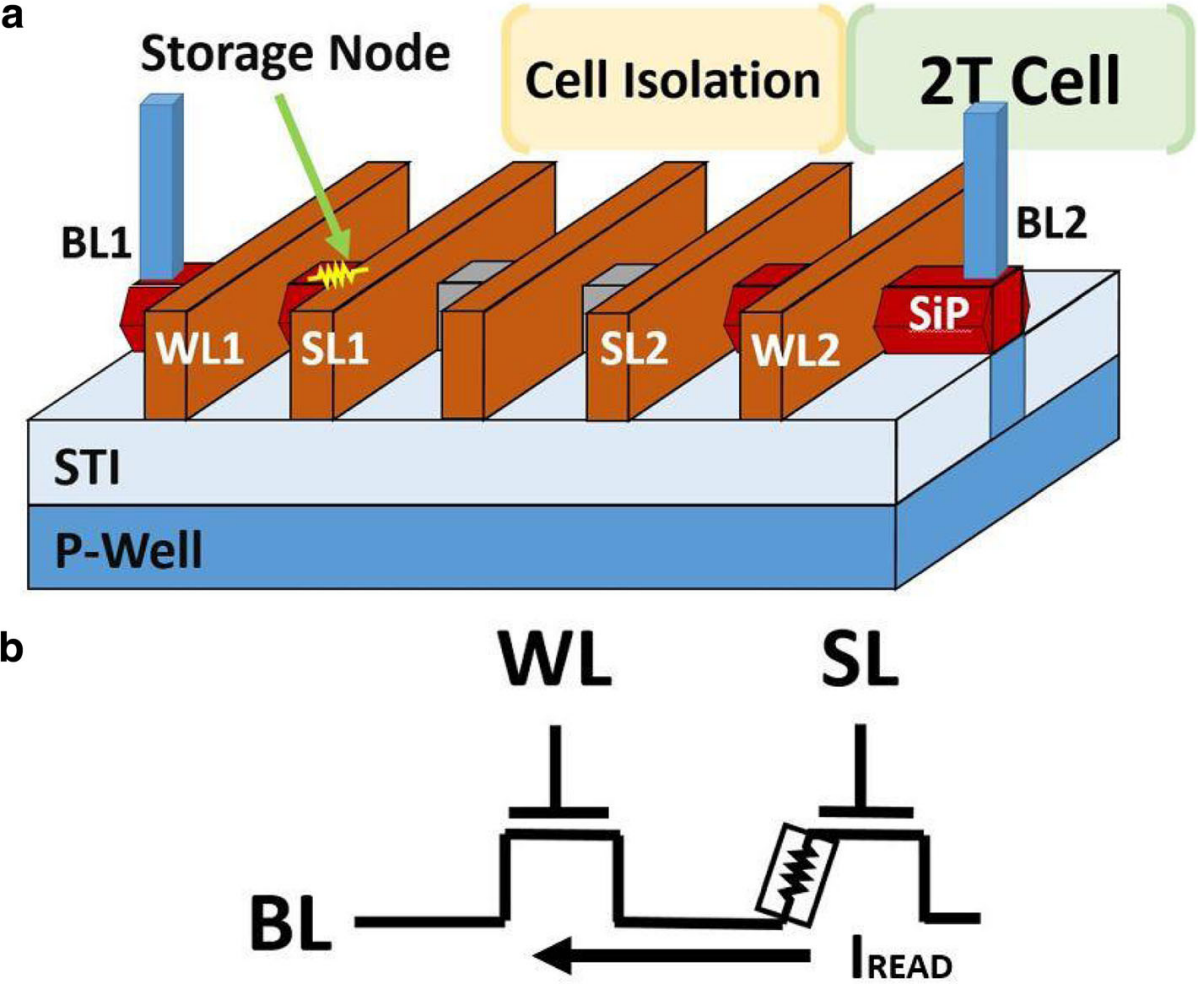
**a** 3D illustration of the 2 T FIND RRAM cells implemented by CMOS FinFET technologies and **b** the circuit schematic of a unit cell for FIND RRAM under read condition is shown, where VSL = VWL = 0.8 V and BL is biased at zero

**Fig. 2:**
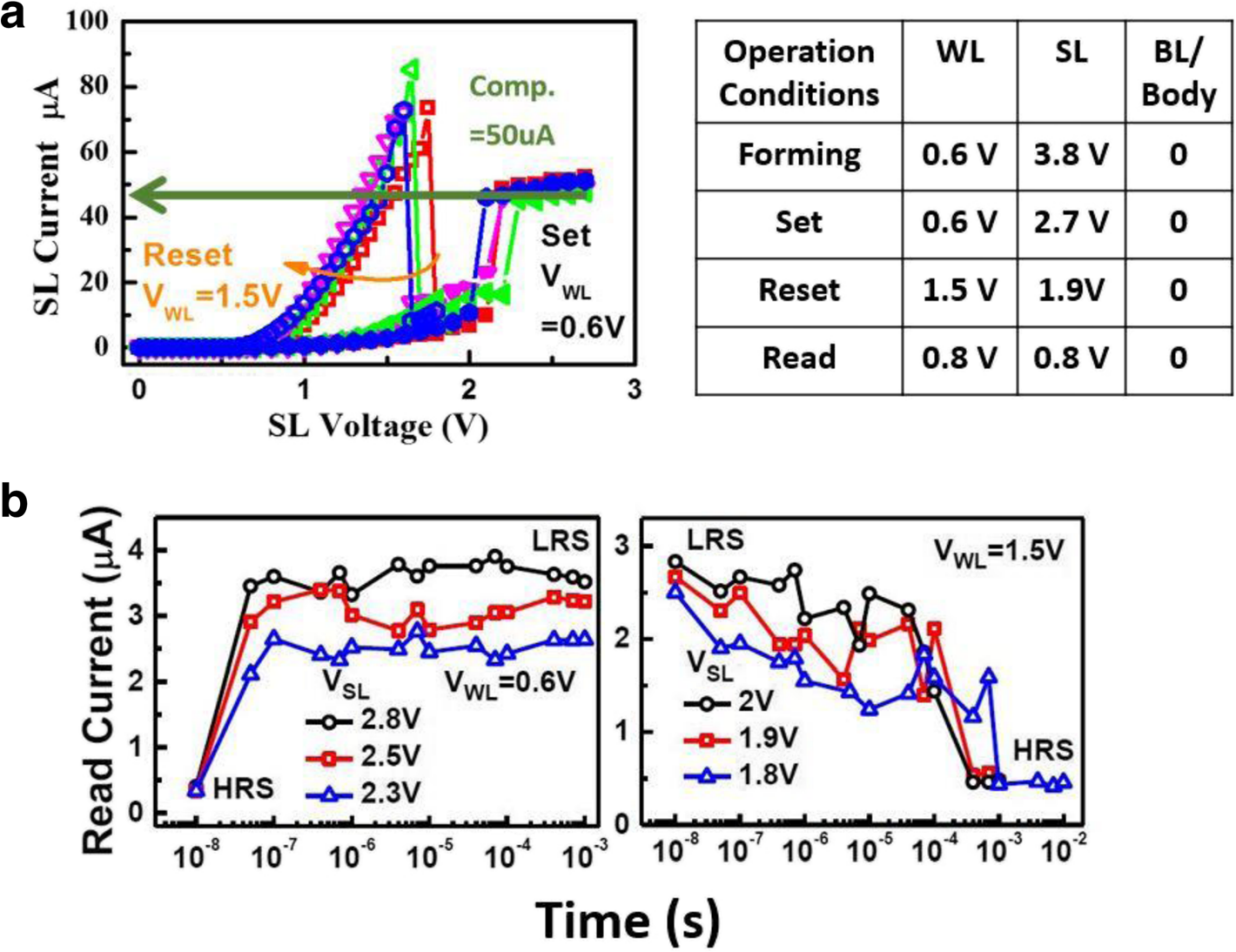
The DC resistive switching features of the FIND RRAM and its operation conditions is shown in (**a**). Its time-to-set and time-to-reset characteristics are summarized in (**b**)

**Fig. 3:**
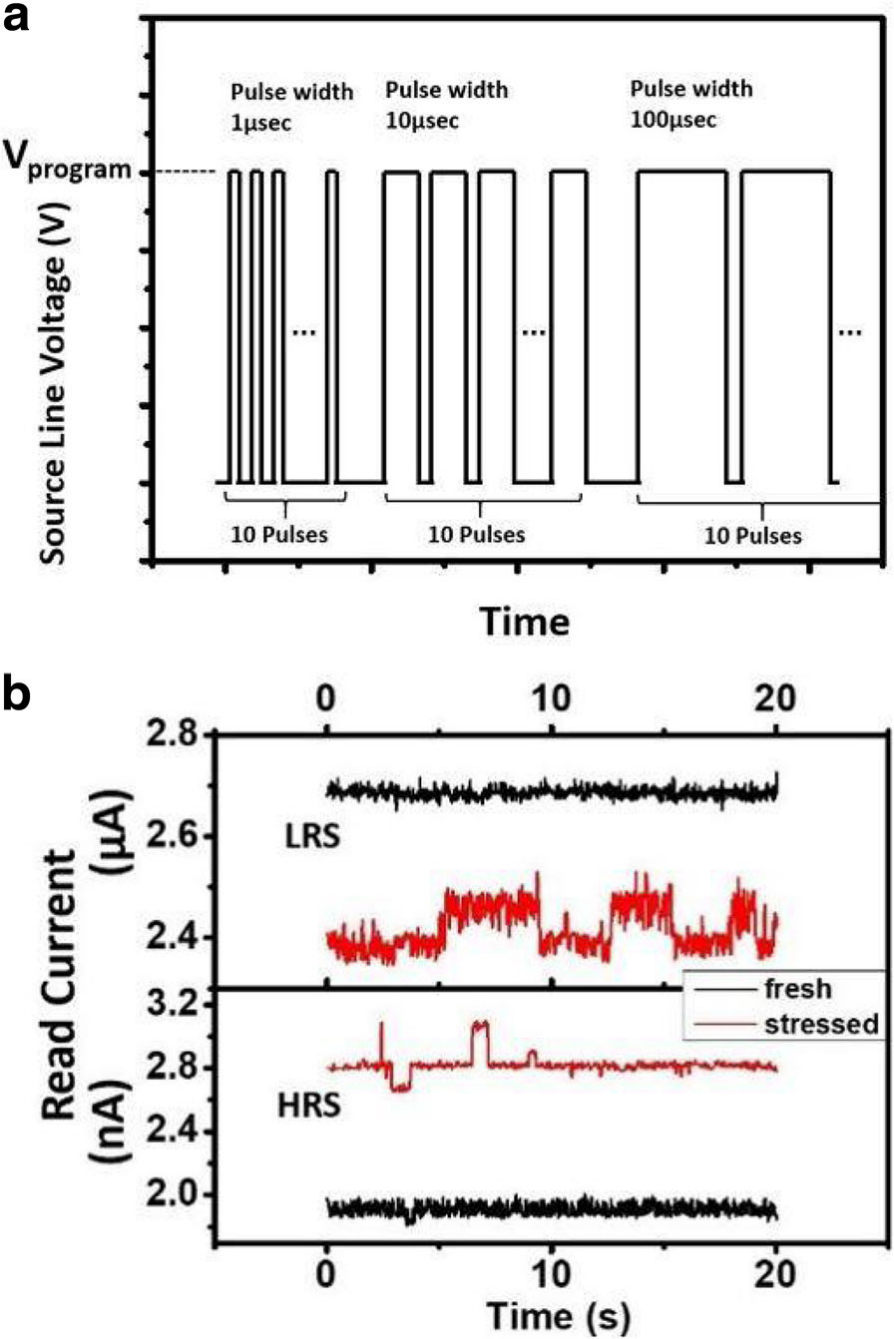
**a** Illustration of the increasing pulse width used to perform set/reset operation. After the first pulse of 1 μs, a read operation is performed to evaluate whether the read current reach the target levels of 3 μA for LRS, and less than 1 μA for HRS. If the required state is not achieved, the next pulse is given. The pulse width is extended by an order every ten pulses. **b** Comparison of read current at room temperature sampled at VSL = VWL = 0.8 V and VBL = 0, with sampling rate of 500 Hz, before and after being cycled 100 times

A FIND RRAM consists of two FinFET transistors. One act as the WL select transistor in series of the RRAM node. The high-k gate dielectric between the SiP and SL of the other transistor serves as a storage node, as shown in Fig. 1a. The FIND devices consist of the gate dielectric film in standard FinFETs, where the gate electrode is W for the top electrode and TaN for the bottom. The gate stack oxide consists of HfO_2_/SiO_2_/TiN stack layers [12]. The read condition of a FIND RRAM cell is illustrated in Fig. 1b, where WL is given 0.8 V to turn on the select transistor, while 0.8 V is added to SL in order to drive a sufficient read current for data read out. Resistive switching between high resistance state (HRS) and low resistance state (LRS) states is achieved by performing set/reset on the FIND RRAM cell [13]. A FIND RRAM shows fairly stable resistive switching features under DC sweeps, see Fig. 2a, while its time-to-set and time-to-reset characteristics are summarized in Fig. 2b. The operation conditions listed in Fig. 2a show that low-voltage operations are possible. The cells undergo multiple pulse cycles for both set and reset in order to reach the targeted read current levels. Pulse width will increase if the device fails to set or reset after multiple pulses are applied, as illustrated in Fig. 3a. Distinctive RTN signal can be found in both LRS and HRS FIND RRAM cells, as shown in Fig. 3b. RTN signals caused by charge trapping and detrapping in the current conducting path can lead to significant current fluctuation [14–17].

It is well established that repeatedly cycling, i.e., switching between the two states, can induce stress on the gate dielectric layering FinFET [18]. The stressed transition metal oxide (TMO) layer in the FIND RRAM cells has a stronger tendency to show RTN noises, leading to time-variant read current which can cause read error and stability challenges during data read out. In this experiment, we sampled the read currents of the array at fresh, after 10× cycles and after 100× cycles, in order to observe the stress effect on RTN in the FIND RRAM.

To investigate the temperature effect on stressed FIND RRAM cells, samples with distinct RTN signals in LRS are first cooled down to 0 °C, then, gradually heated it up to 50 °C. During this process, read currents at these temperatures are sampled continuously for 20 s as a rate of 500 Hz. This gives us some clue as to how RTN behave under temperature change.

## Results and Discussion

**Fig. 4:**
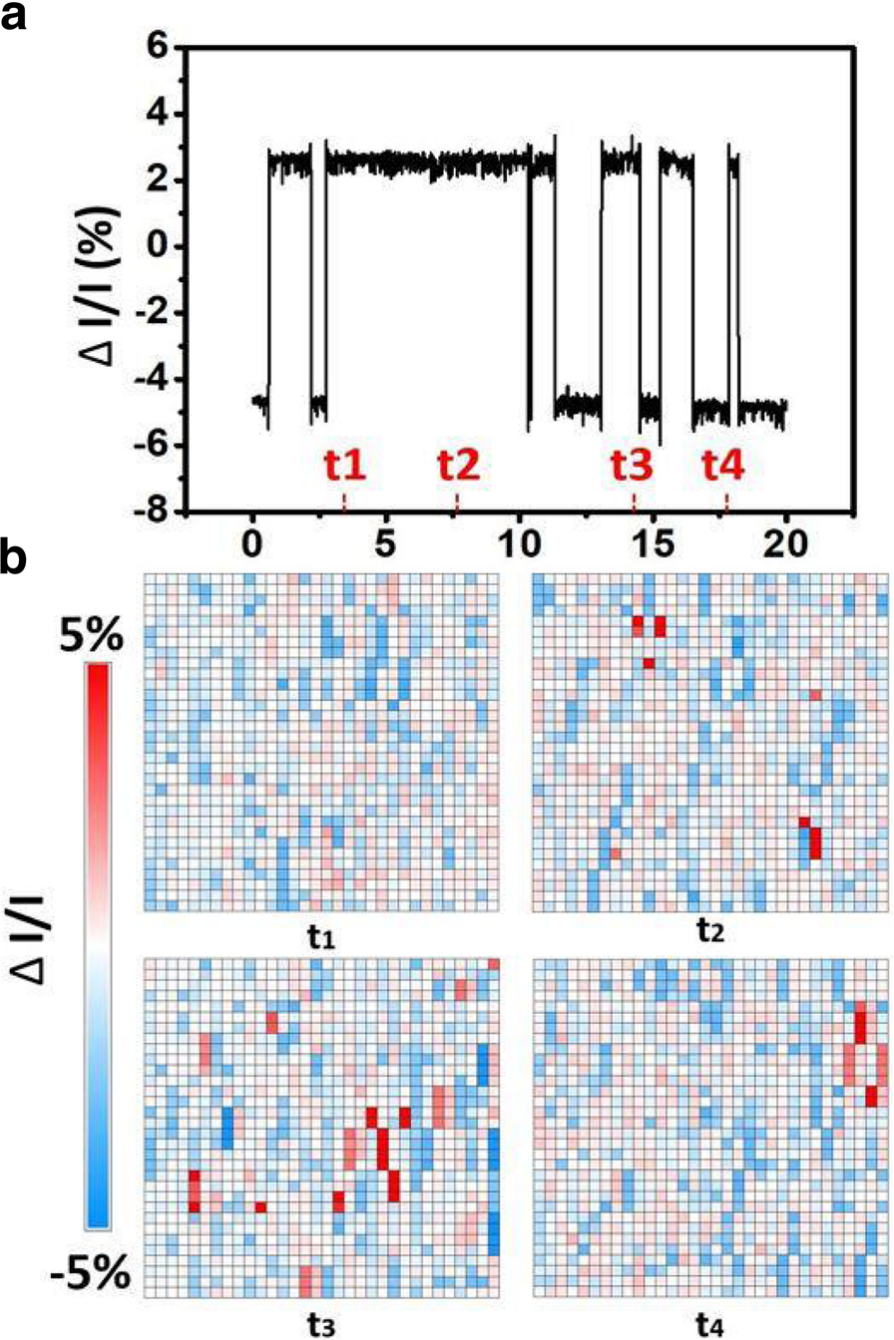
**a** A normalized read current at 25 °C sampled at a rate of 500 Hz in a RRAM cell that had gone through 10 cycles. This shows that RTN causes time-variant read current. **b** Shows the plot of normalized current in 1kbit array that had gone through 10 cycles, sampled at simultaneously at t1 = 3, t2 = 7.5, t3 = 14, and t4 = 17.5 s, sequentially. By comparing the snapshot at different time, read current fluctuates under the same read conditions

**Fig. 5:**
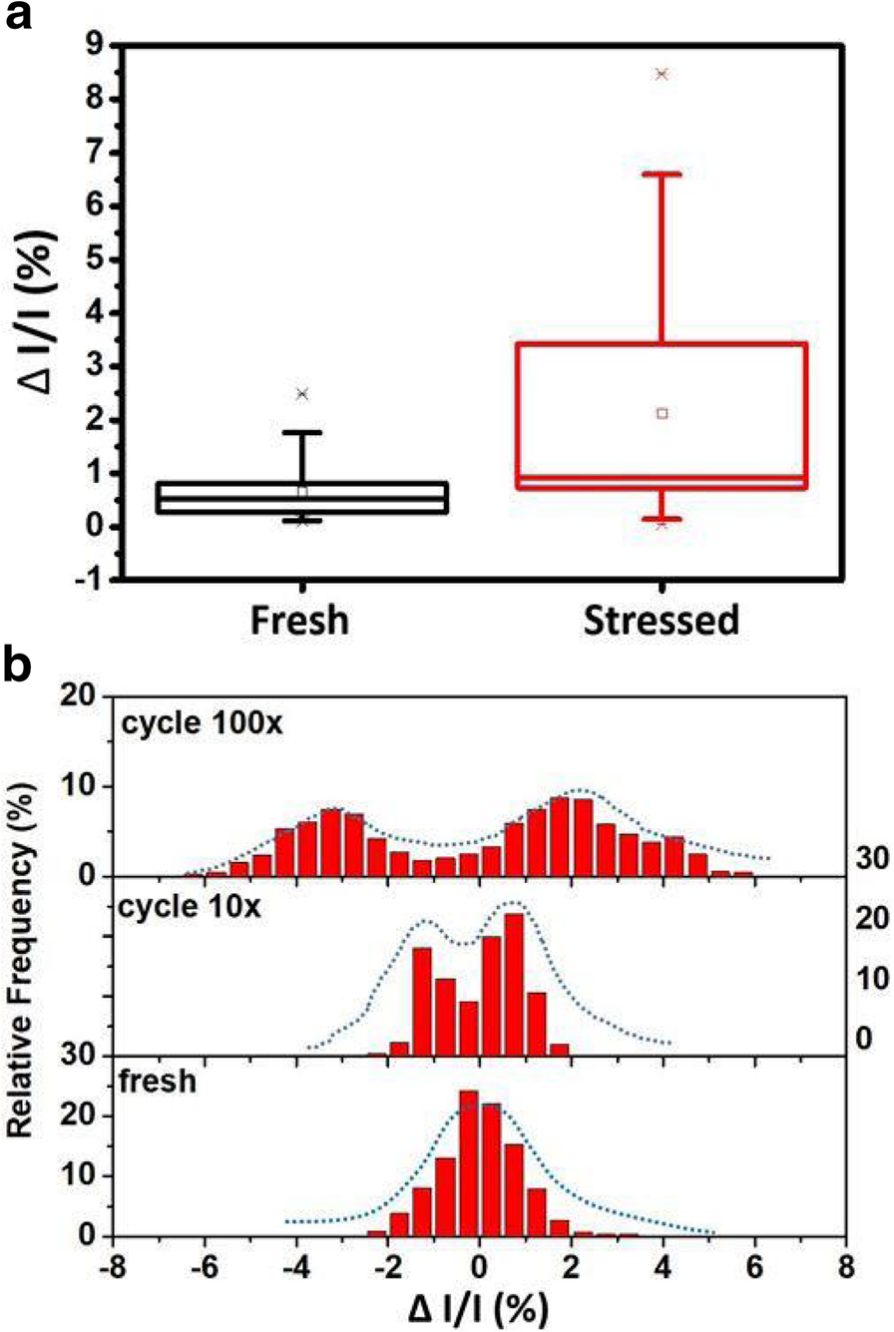
**a** Comparing the distribution of the |ΔI/I| of 50 samples before and after stress. It can be seen that cells exhibit overall larger ΔI/I after stress. The percentile values of the box plot from bottom to top represent the 25, 50, and 75 percentile points, respectively, while the whiskers indicates the maximum and minimum values. **b** A histogram of normalized read current distribution of a cell measured at fresh and after 10 and 100 cycles. This shows that once a FIND RRAM undergoes long cycling stress, its bit cell current can be subject to more drastic fluctuation due to the addition of RTN

Through extensive measurement, it is found that RTN is more stable and easily observable in a FIND RRAM cell at its LRS. Therefore, in the study of cycling and annealing effect on RTN of FIND RRAM cells, the following section focuses on investigating cells at LRS. As shown in Fig. 4a, RTN noises leads to time-variant read current which causes read error. This effect can be observed in the 1kbit cell array. In an array that had gone through 10 cycles, significant read current fluctuations are found when sampling the read current during a 20-s interval. The normalized bit current map in an array is arranged in Fig. 4b, where the current fluctuations in LRS can be as high as + 5%, which is comparable to the RTN observed in gate leakage current after stress [19]. To investigate the effect of stress, we tracked the current fluctuation levels of 50 cells before and after cycling stress. Data in Fig. 5a reveals more than 90% of the cells exhibit an increase in ΔI/I after cycling. Namely, the time-variant noise in the FIND RRAM is shown to be worsened gradually as the cells experienced more cycling stresses. As we compare the normalized read current distribution of fresh cells and cycled cells, it is found that cells which gone through more cycles exhibit more significant RTN signals, and show two or more peak current distributions at particular states. On the other hand, fresh cells have a current distribution of a standard distribution, hinting that there are no RTN noises involving in the fluctuation, as demonstrated in Fig. 5b [20, 21]. This suggests that once a FIND RRAM undergoes long cycling stress, its bit cell current can be subject to more drastic fluctuation due to the addition of RTN.

**Fig. 6:**
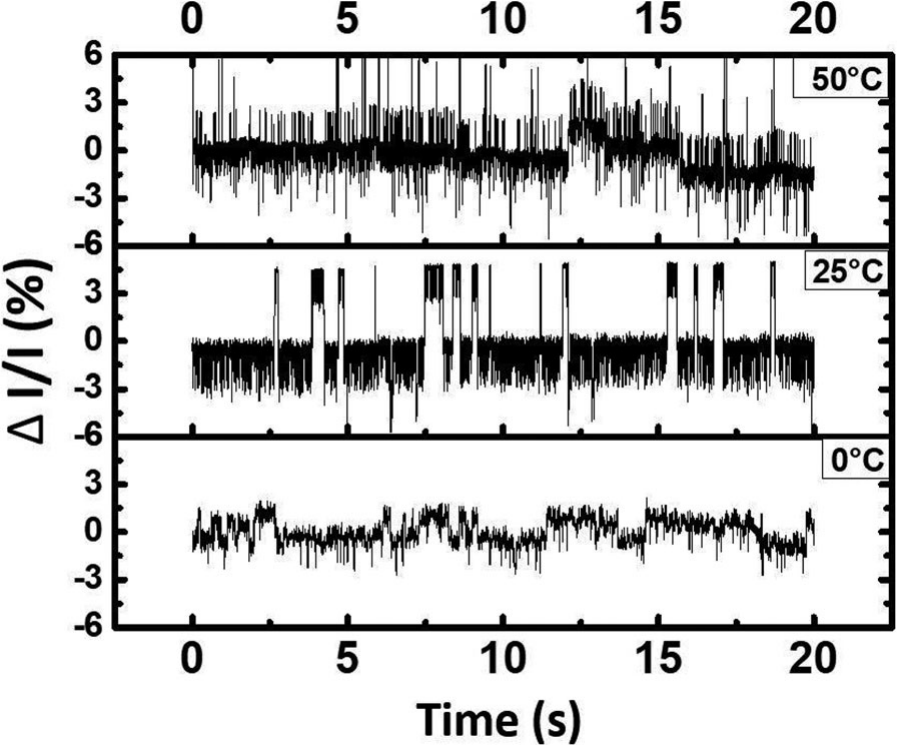
A comparison of normalized read currents of a RRAM cell (in LRS state) that had gone through 100 cycles with RTN noise measured at 0, 25, and 50 °C, at a sample rate of 500 Hz

**Fig. 7:**
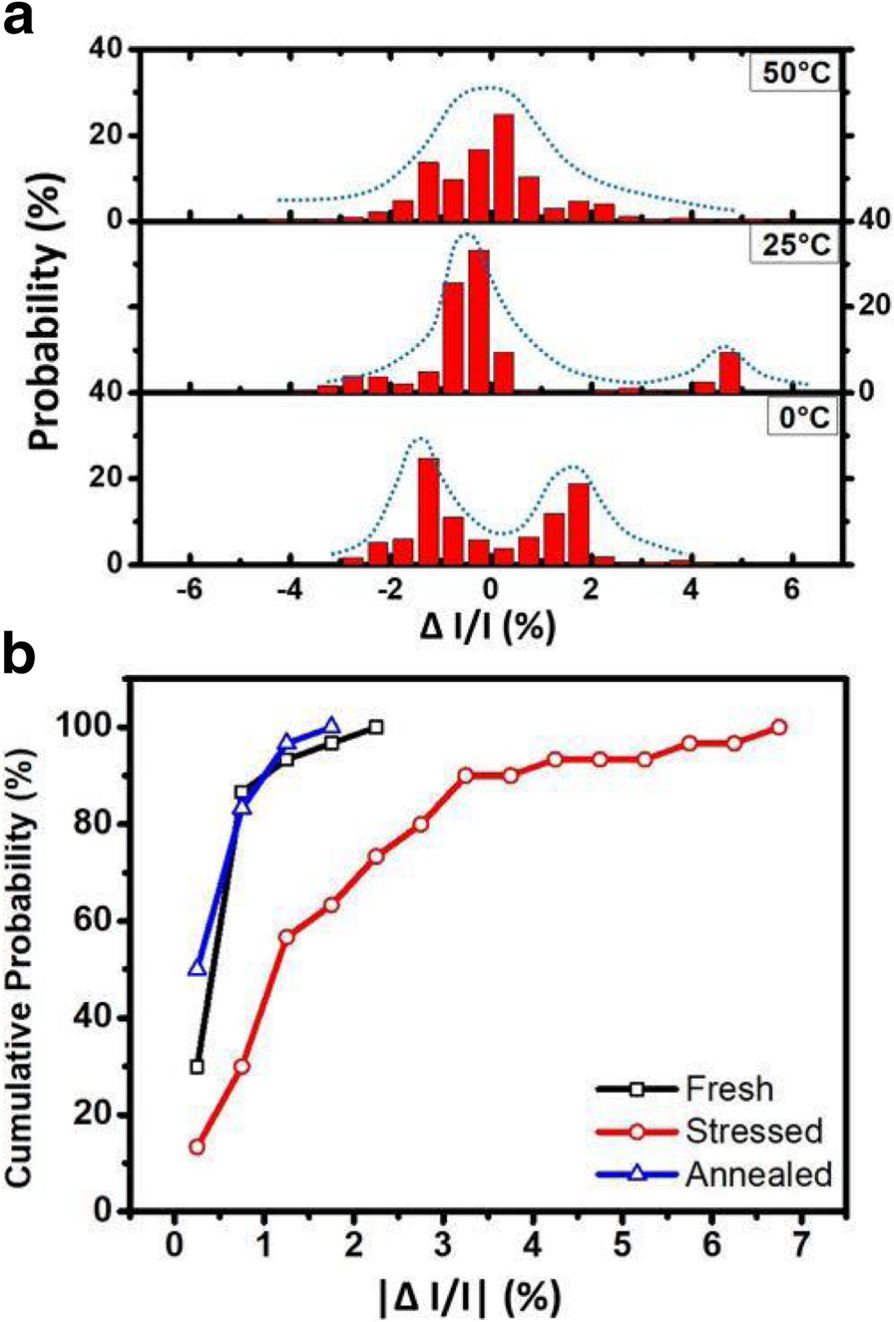
**a** A comparison of histograms of normalized read current distribution of a cell in LRS state measured at 0, 25, and 50 °C. **b** A comparison of the cumulative probability of the |ΔI/I| of 30 samples before stress, after stress and after annealed, respectively. It is found that a large portion of the cells return to its original ΔI/I after annealing process

The normalized results of the read current sample at different temperature stages are compared in Fig. 6. The capture time and emission time of RTN traps have been studied and are known to change with temperature, both of which decrease as temperature increases [22, 23]. As expected, the frequency of RTN noise raises as temperature increases, namely that the fluctuations of read current at 0 °C occur less frequently than those at 25 °C. However, when measured at 50 °C, current fluctuations between two states become less prominent. This can be further revealed in Fig. 7a, which plots the histogram of normalized read currents measured at 0 °C, 50 °C, and room temperature. The current distributions of 0 °C and 25 °C do have two distribution peaks, suggesting single trapping states RTN dominating the conductive path [19], while the discrete states on the sampled current at 50 °C become less prominent. This suggests that at higher temperature, the traps which cause the RTN signal might subject to instability like annealing effect or defect recombination, consequently affecting the electron trapping probability, and thus easing the RTN effect [27].

**Fig. 8:**
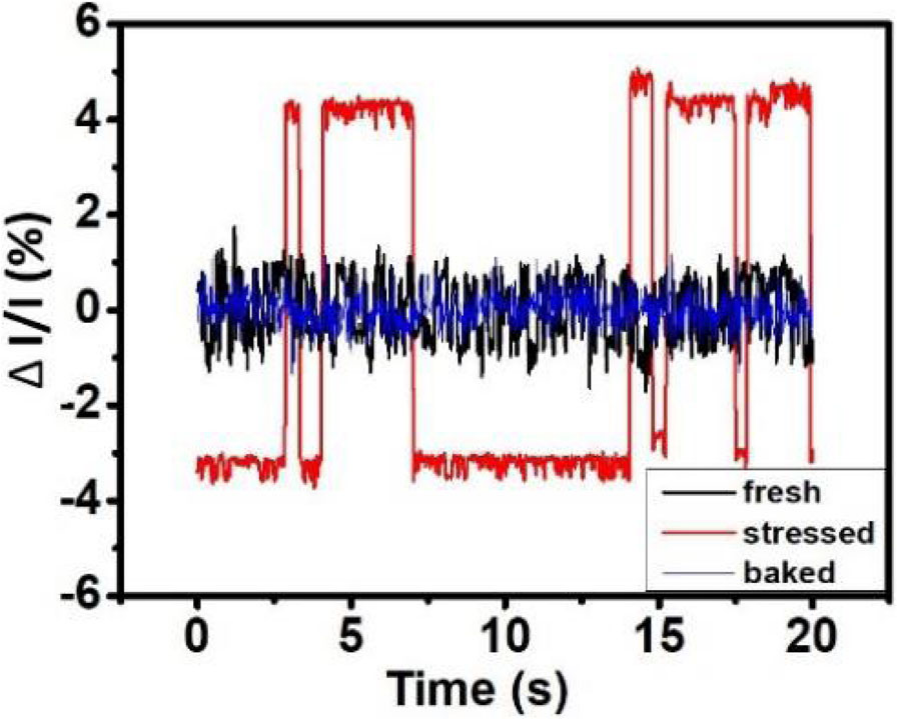
Read currents of fresh and stressed cells sampled at 25 °C, 500 Hz compared to that of the cell after high temperature bake and cooled down in a period of 30 min to room temperature. It shows that after the bake, the stress is relieved and the cell behaves similar to a fresh one

**Fig. 9:**
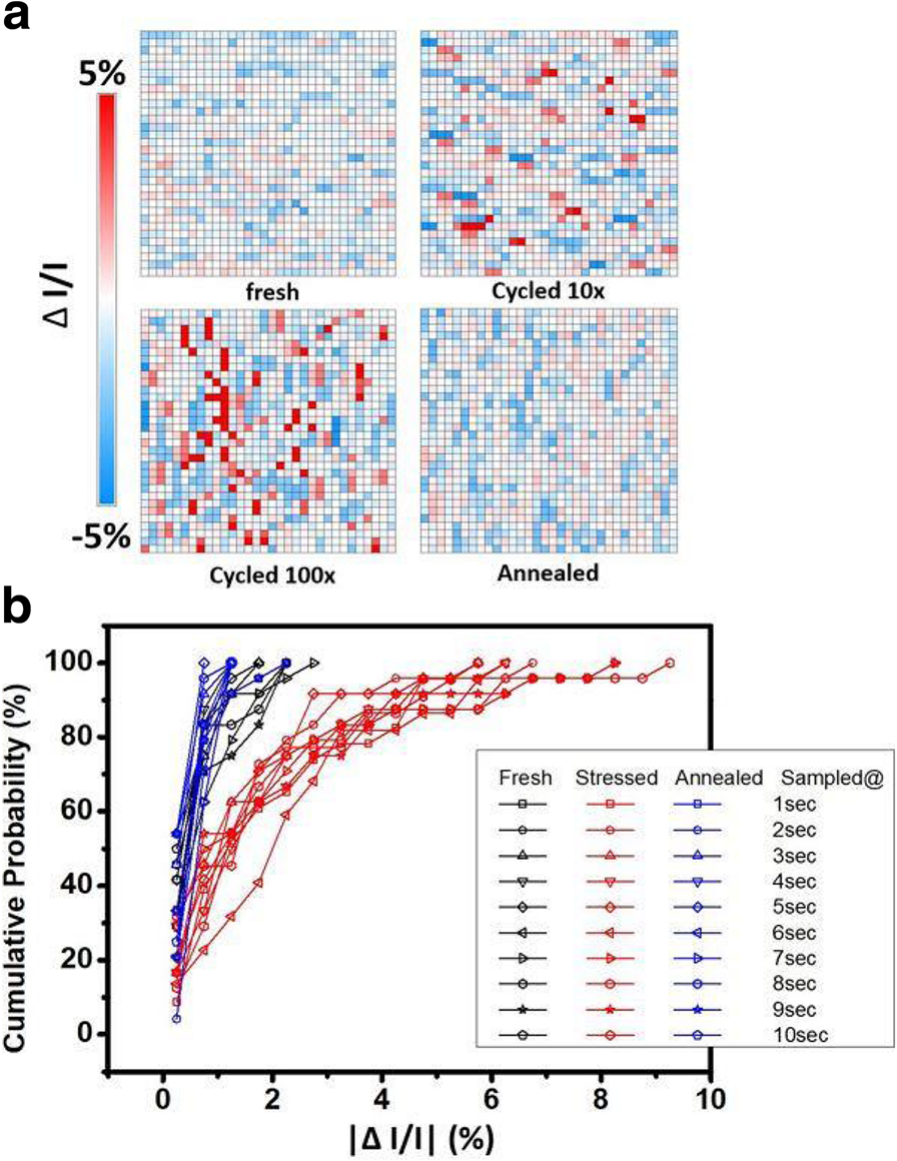
**a** Normalized read currents of a 1kbit array taken at a specific time plotted at fresh, lightly stressed, highly stressed and after annealed. It could be seen that number of cells with strong fluctuation greatly increases across the array as the array undergoes more stress, and dies down after it is annealed. **b** Comparison of the cumulative probabilities of the |ΔI/I| distributions of 50 samples from the same array in its fresh, stressed and after annealed states, at different time instances with a 1-s interval. The overall ΔI/I on samples after stress is higher, and it returns back to normal after annealing

In further study, samples are heated up to 75 °C. These cells cooled down to room temperature after 30 min, then the samples are taken. Thirty cells are chosen and their cumulative current fluctuation levels at fresh, stressed, and after annealed are compared in Fig. 7b. This shows that most cells return to its original ΔI/I after the annealing process. The normalized read currents from a cell in its fresh state, stressed state, and after baked are compared in Fig. 8. The current after bake resembles the one which is fresh, suggesting that high temperature bake anneals the traps that cause RTN signals. Figure 9a is an arranged plot of the normalized read current for 1kbit array of fresh cells, slightly stressed cells, highly stressed cells, and that after bake, respectively. From the plot, it is demonstrated that current fluctuations becomes more intense when the RRAM is highly stressed, and is drastically reduced after the high temperature bake. This effect can be observed in the whole array, which confirms that high-temperature bake does in fact provides an annealing effect to the traps that induce RTN noise [24–29]. Figure 9b further compares the ΔI/I distributions in a cell array obtained by ten different sampling sequentially. It is found that the same array exhibits different ΔI/I distributions in its fresh, stress, and after annealed states. Data suggested that annealing does helps the cell array to show reduced current fluctuations, similar to the level of an array in its fresh state. This result can be used to correct and extend the lifetime of FIND RRAM cells which have exhibited RTN signals after cycling stress.

**Fig. 10:**
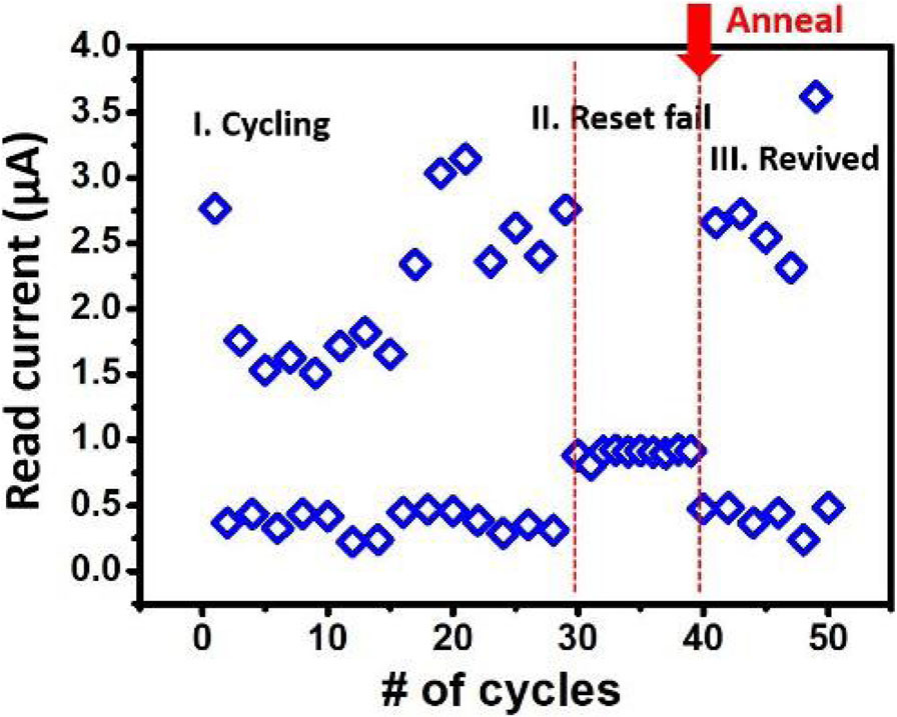
By applying 125 °C bake to cells that are stuck in middle state and providing an annealing process, we convert these cells back to functional state, thus prolonging the cell lifetime

**Fig. 11:**
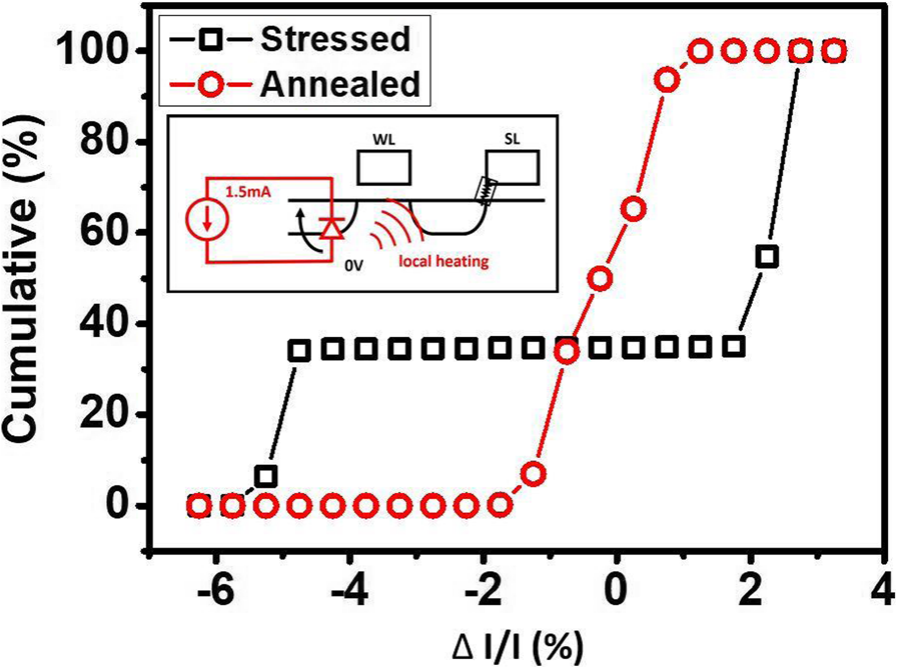
The on-chip annealing scheme we proposed involves applying − 1 V at BL resulting forward bias and a measured current of 1.5 mA, which heats up and anneals the stressed cell. In the plot, by comparing the cumulative percentage of the normalized current of the cell before and after the process, we can see that the current fluctuation caused by RTN is greatly reduced

It is worthy of mentioning that some cells stuck in a middle state during cycling are revived after high-temperature treatment. As a FinFET RRAM cell cycles through LRS and HRS, the conductive filament in some cells may form a channel that cannot be reset easily. The bake process provides those cells path to redistribute its oxygen vacancies, consequently allowing an effective reset to HRS [30]. Figure 10 shows a cell which was not able to switch to HRS being revived after 125 °C, 15 min bake. With this in mind, an on-chip annealing mechanism involving heating up the FIND RRAM cell locally is proposed. A constant current of 1.5 mA with a period of 10 s is applied to the cell through forward bias at the drain junction of the select FinFET, as illustrated in Fig. 11a. This large current heats up a confined region near the RRAM TMO, and provides a similar annealing effect. The read current before and after annealed of the device under test (DUT) is measured and analyzed in Fig. 11b. The cumulative distributions of the continuously sampled read current demonstrate the removal of RTN on a cell after on-chip anneal step. Here, the on-chip annealing of the FIND RRAM was performed on single cells in a sequential steps to understand the nominal response to a typical RRAM cell after stress. New on-chip annealing procedure needs to be developed for an array-level experiment.

## Conclusion

In this paper, the stress and temperature effect on RTN in FIND RRAM cell array is discussed. Cycling stress-induced RTN increase is observed. Effect of high-temperature treatment on reducing RTN and relieving stress for TMO in a FIND RRAM is observed. Finally, an on-chip annealing scheme is proposed and demonstrated.

## Abbreviations

FIND RRAM: Fin field-effect transistor dielectric resistive random access memory
HRS: High resistance state
LER: Line-edge roughness
LRS: Low resistance state
RTN: Random telegraph noise
TMO: Transition metal oxide
